# Oxidative balance score was negatively associated with the risk of metabolic syndrome, metabolic syndrome severity, and all-cause mortality of patients with metabolic syndrome

**DOI:** 10.3389/fendo.2023.1233145

**Published:** 2024-01-12

**Authors:** Zhixiao Xu, Xiong Lei, Weiwei Chu, Luoqi Weng, Chengshui Chen, Ran Ye

**Affiliations:** ^1^ Department of Ultrasonography, The Second Affiliated Hospital and Yuying Children’s Hospital of Wenzhou Medical University, Wenzhou, China; ^2^ Department of Pulmonary and Critical Care Medicine, The First Affiliated Hospital of Wenzhou Medical University, Wenzhou, China; ^3^ Department of Pulmonary and Critical Care Medicine, The Lu’an People’s Hospital of Anhui Province, The Lu’an Hospital Affiliated to Anhui Medical University, Lu’an, China; ^4^ Key Laboratory of Interventional Pulmonology of Zhejiang Province, The First Affiliated Hospital of Wenzhou Medical University, Wenzhou, China; ^5^ The Quzhou Affiliated Hospital of Wenzhou Medical University, Quzhou People’s Hospital, Quzhou, China

**Keywords:** oxidative stress, metabolic syndrome, lifestyles, diet, mortality

## Abstract

**Background:**

The oxidative balance score (OBS), an encompassing scoring mechanism for assessing oxidative stress, is formulated based on nutritional and lifestyle components. The emergence of metabolic syndrome (MetS) is intricately linked to oxidative stress. Nonetheless, the correlation between OBS and MetS displays variability within distinct cohorts.

**Objective:**

We worked on the relationships between OBS and the risk of MetS, MetS severity, and all-cause mortality of MetS patients.

**Methods:**

A total of 11,171 adult participants were collected from the U.S. National Health Examination Survey (NHANES) 2007-2018. Employing survey-weighted logistic models, we evaluated the relationship between OBS and MetS risk. Furthermore, survey-weighted linear models were utilized to investigate the connection between OBS and MetS severity. Among the participants, 3,621 individuals had their survival status recorded, allowing us to employ Cox proportional hazards regression models in order to ascertain the association between OBS and the all-cause mortality within the subset of individuals with MetS. The OBS (where a higher OBS signified an increased prevalence of anti- or pro-oxidant exposures) weighed the 20 factors, while the MetS severity score weighed the five factors.

**Results:**

After multivariable adjustment, individuals with elevated OBS were found to exhibit a decreased susceptibility to MetS [odds ratio (OR) 0.95; 95% CI 0.94-0.96]. The adjusted OR was 0.42 (95% CI 0.33-0.53) for MetS risk in the fourth OBS quartile compared with those in the first OBS quartile (P for trend < 0.001). A one-unit increase in OBS was linked to a 3% reduction in MetS severity score by 3% (mean difference, -0.03; 95% CI, -0.04 to -0.03). Moreover, increased OBS correlated with decreased hazard of all-cause mortality risk among MetS subjects (adjusted hazard ratio, 0.95; 95% CI, 0.93-0.98). These associations retained their strength even subsequent to the introduction of sensitivity analyses. There existed a statistically significant negative correlation between diet/lifestyle OBS and both MetS risk as well as MetS severity.

**Conclusions:**

An inverse correlation was observed between OBS and the susceptibility to MetS, MetS severity, and all-cause mortality of MetS patients. Health outcomes for MetS patients were positively related to antioxidant diets and lifestyles.

## 1 Introduction

Metabolic syndrome (MetS) and its associated disorders, a major risk factor for cardiometabolic complications (1), are responsible for the majority of non-communicable diseases’ deaths (2). MetS, a current silent epidemic disease, is a multi-faceted disorder with a highly complex and unclear pathogenesis (3). Growing evidence backs up that pro-oxidant/antioxidant imbalance plays a decisive role in a cluster of symptoms (4), including hypertension (5), obesity (6), insulin resistance (7), and NAFLD (8). Furthermore, antioxidant therapy has been proposed to have a beneficial effect on the prevention of MetS-related diseases (9–11), even though the benefits of antioxidant supplementation have not been confirmed by many epidemiological studies (12, 13).

Oxidative stress, which is to blame for in the development of many diseases (14), arises from an imbalance stemming from the excessive generation of harmful reactive oxygen and nitrogen species (15). This intricate equilibrium is governed by a complex network of antioxidants (16, 17). In a healthy state, this balance is upheld by the generation of highly reactive derivatives of oxygen metabolism (reactive oxygen species (ROS)) and their elimination by enzymatic/nonenzymatic antioxidants (18). An assortment of dietary (vitamin C, vitamin E, some carotenoids and flavonoids (17)) as well as non-dietary components (cigarette smoke and alcohol (19, 20)) can directly or indirectly contribute to the perturbation of the pro-oxidant/antioxidant equilibrium. Furthermore, different types of nutrient-mediated pro-oxidant/antioxidant imbalance possess the capacity to incite inflammatory responses (21). Notably, in postmenopausal Mexican women, markers of oxidative stress were significantly altered in those with MetS compared to those without, such as a drop in blood vitamin C (22).

An oxidative balance score (OBS) has been conceptualized through a series of observational investigations as a means to encompass the cumulative oxidative impacts arising from diverse exposures to pro-oxidants/antioxidants (23, 24). An OBS is constructed by aggregating scores based on quantiles or categories pertaining to dietary/lifestyle exposures. Moreover, the components included in this study were all discerned and validated within the framework of the National Health and Nutrition Examination Survey (NHANES) (23, 24), rendering them notably comprehensive within the ambit of this present study. Furthermore, the interaction between OBS and many diseases was evaluated, such as diabetes (25) and cardiovascular disease (26). However, the relationship between OBS and MetS risk has not been consistently demonstrated in all cohorts. For example, a small population-based study of Tehranian adults failed to confirm a remarkable correlation between OBS and the risk of MetS (27). Although the OBS approach is promising, the lack of consistent findings across different populations suggests that additional research is required to refine the methodology and establish its validity in diverse contexts.

Maximizing the comprehensiveness of OBS components and utilizing data from the U.S. National Health and Nutrition Examination Survey (NHANES) for analysis could enhance the precision of this correlation. The aim was to delve deeper into the potential connections between OBS (including dietary OBS and lifestyle OBS) and the susceptibility of MetS, MetS severity, and all-cause mortality of MetS patients. It was posited that oxidative stress was essential in the context of MetS, and there existed a conjecture regarding the potential variability of the OBS-MetS relationship across diverse populations. This investigation was carried out within the framework of the U.S. NHANES dataset.

## 2 Research design and methods

### 2.1 Study population

**Figure 1:**
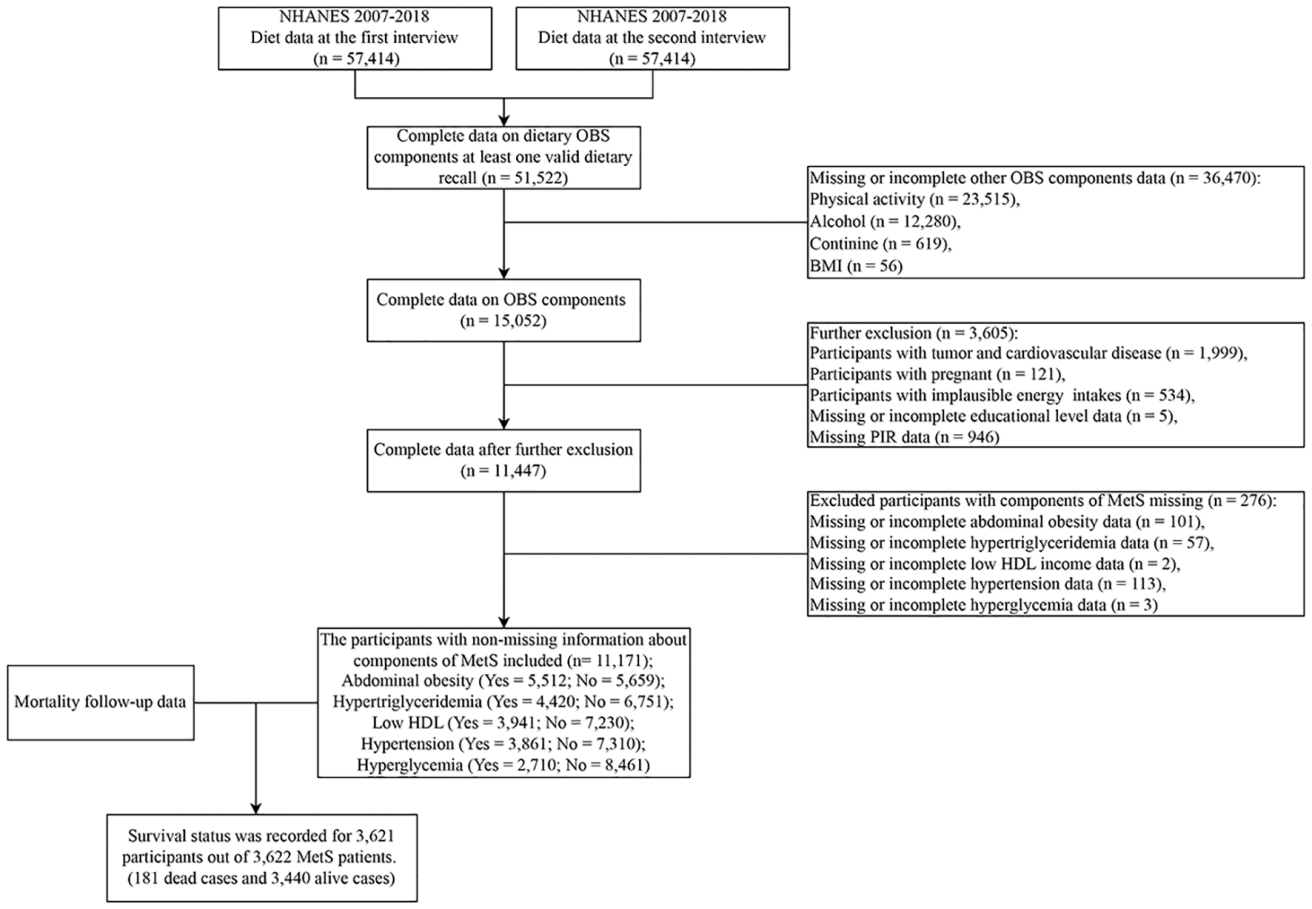
Flowchart of population included in our final analysis, U.S. National Health and Nutrition Examination Survey (NHANES), 2007-2018. OBS, oxidative balance score; MetS, Metabolic syndrome.

Participants in NHANES (2007-2018) were employed. Participants without even one valid diet recall, participants without information on OBS components, participants with tumors and cardiovascular disease, pregnant women, participants with implausible energy intakes (<500 kcal/day or >3,500 kcal/day for females and <800 kcal/day or >4,200 kcal/day for males) (23, 24, 28), participants without information on covariates (educational level and poverty income ratio (PIR)), and participants without information components of MetS were excluded. Eventually, 11,171 subjects were included (**Figure 1**).

Utilizing a sophisticated sampling framework, the NHANES allows for the extrapolation of estimations to the broader spectrum of the U.S. populace. The NHANES includes interviews and examinations (29, 30). Ethical clearance for the execution of NHANES was obtained from the Ethics Review Board of the National Center for Health Statistics Moreover, comprehensive written informed consent was diligently obtained from each participant.

### 2.2 Main exposure

The main exposure was the OBS. Four lifestyle variables and sixteen nutrients were combined to create the OBS, and the information was described in **Table S1**. The 20 components in this study-five prooxidants and fifteen antioxidants (23, 24)-represented the pertinent variables identified in the NHANE. It is acknowledged that divergent datasets utilized in alternative studies may encompass a slightly disparate array of factors (27, 31) (**Table S2**). Nonetheless, given due consideration to the distinctive attributes of NHANES and a thorough review of antecedent investigations, our selection of factors is deemed comprehensive and reflective of the prevailing research landscape. Variables were categorically scored from 0 to 2 based on the sex-specific tertile of each component, and the point assignment for antioxidants and prooxidants was inverse. To enable generalization of the results, the calculation of the tertiles for each component was weighted. Then, the total score was calculated by the sum of the points for each of the 20 OBS components, and the possible OBS values were on a scale of 0 to 40. Moreover, the higher OBS indicated predominantly antioxidant exposures and lower OBS represented predominantly pro-oxidant exposures.

The sixteen nutrients were derived from the average nutrients’ intake during the 2 days. The two dietary interviews were conducted sequentially, in person and by telephone, respectively. We could obtain at least one 24-hour dietary recall of the participant and the participants’ dietary information from the U.S. Department of Agriculture Food and Nutrient Database for Dietary Studies.

The metabolic equivalent (MET) scores, which were calculated from data collected by the Physical Activity Questionnaire (PAQ), were assigned for physical activity. MET serves to quantify the relative energy expenditure associated with various activities. Within the PAQ survey, the MET values corresponding to distinct categories of activities, encompassing vigorous work-related takes, moderate work-related takes, walking or bicycling for transportation, vigorous leisure-time physical activities, and moderate leisure-time physical activities, were meticulously documented. Moreover, the quantification of physical activity was ascertained through the computation of the product derived by multiplying the MET value by the weekly frequency and duration of each specific physical activity (32).

Alcohol consumption was collected from the Alcohol Use Questionnaire, the average amount (drinks) of alcoholic beverages on those days when alcohol was consumed in the past 12 months was treated as sex-specific tertiles and subsequently assigned points. Body mass index (BMI) was from NHANES body measures collected by trained health technicians. Cotinine is generally regarded as the marker of active smoking and secondhand smoke exposure. This is owing to its distinct advantages, including a high concentration and a prolonged half-life. Moreover, the method of measurement of serum cotinine was described in detail in the NHANES website.

### 2.3 Main outcomes

The outcomes were the diagnosis of MetS and each of its components. The criteria of MetS are based on the National Cholesterol Education Program Adult Treatment Panel III (33, 34). Participants considered to be with MetS required the presence of at least three of five components (33). **Table S3** showed the definition of MetS.

The data on blood pressure was collected from the acquisition of a minimum of three successive readings during blood pressure measurements. These readings were subsequently computed to derive the arithmetic mean. Details concerning the anti-hypertensive treatment and the diagnosis of hypertension were collected from the blood pressure/cholesterol section in the interview data. Similarly, information regarding anti-diabetic drugs, taking insulin, and the diagnosis of diabetes were meticulously collated from the interview data’s diabetes section.

However, although previous studies have reported several methods to quantify MetS, there is still no validated tool. We chose a continuous MetS severity score developed by Gurka and colleagues (35), because it has been applied in other NHANES studies (36). The MetS severity score weighs the five factors in a way that takes into account gender and race/ethnicity differences in risk status. Operationalized definitions in NHANES have been documented in other studies (36). Moreover, the Homeostatic Model Assessment for insulin resistance (HOMA-IR) was applied to reflect the status of patients.

Furthermore, mortality follow-up data spanning from the date of survey participation through December 31, 2019, were compiled by leveraging information from the National Death Index data (https://www.cdc.gov/nchs/data-linkage/mortality-public.htm).

### 2.4 Covariate definitions

Potential covariates included demographic data, such as age (years), gender (male, female), race/ethnicity (non-Hispanic White, non-Hispanic Black, Mexican American, and other), educational level (<high school, high school/general educational development, and >high school), PIR, and dietary energy intake.

### 2.5 Statistical analyses

Data on demographic characteristics, dietary intake, and outcomes (continuous variables) were shown as the median (P25, P75), while categorical variables were represented as numbers (percentages). The Kruskal-Wallis test was employed for the comparison of continuous baseline characteristics, as the continuous variables analyzed in this study were determined to exhibit non-normal distribution, and the Rao-Scott chi-squared test was adopted to test for categorical variables.

Survey weights from full sample 2-year interview weights (WTINT2YR) were used as recommended by NHANES (1/6 * WTINT2YR), and weighted models were conducted in the following regression analyses. Logistic models were conducted to estimate associations of OBS with the presence of MetS (dichotomous outcomes), the OBS was also categorized into quartiles and served as a categorical variable. Furthermore, linear models were performed to estimate the relationships of OBS with continuous outcomes (HOMA-IR and MetS severity score). Three models were conducted: Model one was the crude model; Model two adjusted for age, gender, race/ethnicity, educational level, and PIR; Model three further adjusted for dietary energy intake based on Model two. These covariates were chosen because they were regarded as clinically relevant confounders of the relationship between OBS and MetS. Moreover, weighted Cox proportional hazards regression models were employed to ascertain and quantify the potential associations of OBS with all-cause mortality among MetS participants.

Analysis was performed in R 4.1.1. P for trend was calculated by converting categorical variables to continuous variables. P for interaction was also calculated. Statistical significance was defined at a two-sided P value < 0.05.

### 2.6 Sensitivity analysis

To estimate the robustness of the results, some sensitivity analyses were undertaken. 1) Stratified analyses by stratifying factors including gender, age, education level, and PIR were performed. 2) The P for interaction was carried out. 3) Examination of the relationships based on dietary OBS and lifestyle OBS. 4) The analysis of removing one component from the total score at one time was conducted.

## 3 Results

### 3.1 Baseline characteristics

Among 11,171 participants, a total of 3,621 subjects were identified as having MetS. The distribution of OBS exhibited statistically significant variations across demographic parameters such as age, gender, race, and education level (P<0.05), as delineated in **Table S4**. Notably, individuals afflicted with MetS demonstrated comparatively lower OBS scores (19 [14, 25]) in contrast to their counterparts without MetS (21 [15, 26]). Furthermore, an elevated OBS was discerned among survivors in comparison to individuals who succumbed, underscoring a potential association between OBS and survival outcomes. According to the results, the variations in the studied variables across different quartiles of OBS were revealed in **Table 1**. The prevalence of MetS was observed to be comparatively lower among those in the 4^th^ OBS quartile (OBS ≥26) in contrast to in the other three quartiles. Moreover, individuals exhibiting lower OBS values displayed elevated levels of both the HOMA-IR and MetS severity scores in comparison to those with higher OBS values. Notably, statistically significant differences were detected between the OBS quartile groups for each component of MetS (P < 0.05). Persons in a higher quartile were more prone to have higher age, PIR, energy intake, and educational level. No significant differences in gender across OBS quartiles were observed.

**Table 1:** Characteristics of participants based on quartiles of oxidative balance score, National Health and Nutrition Examination Survey (NHANES) 2007-2018.

	All	Q1	Q2	Q3	Q4	P value
		<14	14 to 20	20 to 26	≥26	
	N=11171	N=2781	N=2883	N=2971	N=2536	
Age (year)	41 [29;53]	38 [27;50]	41 [29;53]	42 [30;53]	42 [31;55]	<0.001
Gender						0.372
Male	6086 (53.1%)	1452 (51.1%)	1607 (53.3%)	1615 (53.8%)	1412 (53.7%)	
Female	5085 (46.9%)	1329 (48.9%)	1276 (46.7%)	1356 (46.2%)	1124 (46.3%)	
Race/ethnicity						<0.001
Mexican American	1697 (8.3%)	386 (8.6%)	434 (8.5%)	499 (8.6%)	378 (7.4%)	
Other Hispanic	1036 (5.0%)	261 (5.4%)	266 (5.0%)	272 (4.8%)	237 (4.9%)	
Non-Hispanic White	4924 (70.4%)	1077 (64.0%)	1248 (69.2%)	1354 (71.8%)	1245 (75.6%)	
Non-Hispanic Black	2161 (9.3%)	797 (15.7%)	591 (10.1%)	459 (7.1%)	314 (5.4%)	
Other Race - Including Multi-Racial	1353 (7.0%)	260 (6.3%)	344 (7.2%)	387 (7.6%)	362 (6.7%)	
Poverty income ratio	3.45 [1.76;5.00]	2.52 [1.25;4.57]	3.31 [1.74;5.00]	3.58 [1.97;5.00]	4.29 [2.29;5.00]	<0.001
Energy (kcal)	2066 [1617;2612]	1545 [1230;1910]	1929 [1540;2342]	2209 [1817;2713]	2566 [2089;3114]	<0.001
Educational level						<0.001
<High school	1878 (10.6%)	617 (15.3%)	553 (12.7%)	446 (9.1%)	262 (6.0%)	
High school/general educational development	2446 (21.4%)	767 (29.2%)	650 (23.0%)	612 (20.2%)	417 (14.4%)	
>High school	6847 (68.0%)	1397 (55.5%)	1680 (64.2%)	1913 (70.7%)	1857 (79.6%)	
Metabolic syndrome						<0.001
No	7549 (69.2%)	1813 (67.3%)	1888 (66.2%)	2004 (69.0%)	1844 (73.7%)	
Yes	3622 (30.8%)	968 (32.7%)	995 (33.8%)	967 (31.0%)	692 (26.3%)	
Abdominal obesity						<0.001
No	5659 (50.0%)	1170 (40.8%)	1416 (47.7%)	1550 (50.4%)	1523 (59.8%)	
Yes	5512 (50.0%)	1611 (59.2%)	1467 (52.3%)	1421 (49.6%)	1013 (40.2%)	
Hypertriglyceridemia						0.020
No	6751 (61.1%)	1693 (61.1%)	1673 (58.2%)	1801 (61.3%)	1584 (63.5%)	
Yes	4420 (38.9%)	1088 (38.9%)	1210 (41.8%)	1170 (38.7%)	952 (36.5%)	
Low high-density cholesterols						<0.001
No	7230 (66.9%)	1702 (63.5%)	1815 (64.0%)	1924 (67.6%)	1789 (71.7%)	
Yes	3941 (33.1%)	1079 (36.5%)	1068 (36.0%)	1047 (32.4%)	747 (28.3%)	
Hypertension						0.008
No	7310 (68.4%)	1765 (68.0%)	1804 (65.2%)	1975 (68.7%)	1766 (71.5%)	
Yes	3861 (31.6%)	1016 (32.0%)	1079 (34.8%)	996 (31.3%)	770 (28.5%)	
Hyperglycemia						0.029
No	8461 (76.3%)	2090 (77.4%)	2143 (74.0%)	2255 (75.8%)	1973 (78.3%)	
Yes	2710 (23.7%)	691 (22.6%)	740 (26.0%)	716 (24.2%)	563 (21.7%)	
Homeostatic Model Assessment for insulin resistance	12.68 [7.97;21.75]	14.85 [9.14;24.90]	13.69 [8.10;23.46]	12.46 [8.18;21.57]	10.92 [6.77;18.23]	<0.001
MetS severity score	-0.10 [-0.69;0.58]	0.08 [-0.45;0.64]	-0.03 [-0.59;0.68]	-0.08 [-0.68;0.61]	-0.32 [-0.90;0.33]	<0.001

Values in brackets were median (P25, P75) for continuous variables, as well as proportions for categorical variables.

### 3.2 Association of oxidative balance score with metabolic syndrome and its components

**Table 2** and **Table S5** summarized the results of correlations between OBS and MetS. The OBS was significantly negatively related to MetS (adjusted odds ratio (OR): 0.95; 95%CI: 0.94-0.96) after full adjustment. Every one-SD increase in OBS was linked with 32% decreased odds of MetS after full adjustment. When OBS was deemed as a categorical variable, there was also a negative correlation between the highest quartile OBS and MetS (adjusted OR: 0.42; 95%CI: 0.33-0.53), after full adjustment, compared with the lowest quartile OBS. The relationship was also observed in Model 1 and Model 2. Moreover, significant associations were also discovered in the 3^rd^ quartile OBS (Model 2 & Model 3) and the 2^nd^ quartile OBS (Model 3). Importantly, the P for trend was <0.001 in all models.

**Table 2:** Association of oxidative balance score with metabolic syndrome risk.

	Model 1		Model 2		Model 3	
	OR (95%CI)	P value	OR (95%CI)	P value	OR (95%CI)	P value
OBS	0.98 (0.97 to 0.99)	<0.001	0.97 (0.96 to 0.98)	<0.001	0.95 (0.94 to 0.96)	<0.001
OBS per SD	0.87 (0.82 to 0.92)	<0.001	0.81 (0.76 to 0.87)	<0.001	0.68 (0.62 to 0.74)	<0.001
OBS category						
Q1	Reference	Reference	Reference	Reference	Reference	Reference
Q2	1.05 (0.89 to 1.23)	0.553	0.94 (0.79 to 1.12)	0.493	0.81 (0.67 to 0.98)	0.027
Q3	0.92 (0.78 to 1.09)	0.353	0.81 (0.67 to 0.98)	0.030	0.62 (0.50 to 0.76)	<0.001
Q4	0.74 (0.61 to 0.88)	0.001	0.63 (0.51 to 0.77)	<0.001	0.42 (0.33 to 0.53)	<0.001
P for trend		<0.001		<0.001		<0.001

Model 1 was a crude model. Model 2 further adjusted for age, gender, race/ethnicity, educational level, and PIR based on Model 1. Model 3 further adjusted for dietary energy intake based on Model 2.

OR, odds ratio; CI, confidence intervals; OBS, oxidative balance score.

Regarding the MetS components, significant negative associations were observed between OBS with abdominal obesity (adjusted OR: 0.93; 95%CI: 0.92-0.93), hypertriglyceridemia (adjusted OR: 0.96; 95%CI: 0.95-0.97), low high-density cholesterols (adjusted OR: 0.96; 95%CI: 0.95-0.97), hypertension (adjusted OR: 0.96; 95%CI: 0.95-0.97), and hyperglycemia (adjusted OR: 0.98; 95%CI: 0.97-0.99). Similarly, the relationships between OBS quartiles and each MetS component were consistently apparent. Remarkably, these relationships were found to be statistically significant within the highest OBS quartile group (all P values for trend < 0.05), as illustrated in **Table S5**.

### 3.3 The relationship between oxidative balance score and metabolic syndrome severity

The continuous MetS severity score, quantified by the Mets Z-score, is exclusively accessible for adult individuals belonging to Hispanic, white, and black adults (35). The adjusted formula can also be applied to Asian populations (37). Importantly, the score was applied in other NHANES studies (36). However, no formula is currently available for the Mexican-American population. Therefore, we excluded Mexican Americans and some incomplete information, and eventually, 4,380 of 11,171 individuals were calculated for the MetS Z-score.

The adjusted linear regression model indicated that, even after accounting for potential confounders, for each one-unit increment in OBS, a person’s MetS Z-score decreased by 3%. The adjusted mean differences (MDs) for MetS were as follows: -0.17 (-0.29, -0.06) for the 2^nd^ OBS quartile, -0.29 (-0.39, -0.20) for the 3^rd^ OBS quartile, and -0.56 (-0.68, -0.44) for the 4^th^ OBS quartile, in comparison to this reference group (participants in the lowest OBS quartile as the reference). These findings suggested a consistent trend of decreasing MetS severity scores with higher OBS quartiles, as supported by a statistically significant trend (P for trend < 0.05) (**Table 3**).

**Table 3:** The relationship between oxidative balance score and metabolic syndrome severity (MetS Z-score).

	Model 1		Model 2		Model 3	
	MD (95%CI)	P value	MD (95%CI)	P value	MD (95%CI)	P value
OBS	-0.02 (-0.03 to -0.02)	<0.001	-0.02 (-0.03 to -0.02)	<0.001	-0.03 (-0.04 to -0.03)	<0.001
OBS per SD	-0.14 (-0.18 to -0.11)	<0.001	-0.15 (-0.18 to -0.11)	<0.001	-0.23 (-0.28 to -0.19)	<0.001
OBS category						
Q1	Reference	Reference	Reference	Reference	Reference	Reference
Q2	-0.07 (-0.18 to 0.05)	0.246	-0.10 (-0.21 to 0.01)	0.073	-0.17 (-0.29 to -0.06)	0.004
Q3	-0.14 (-0.23 to -0.05)	0.002	-0.16 (-0.24 to -0.08)	<0.001	-0.29 (-0.39 to -0.20)	<0.001
Q4	-0.35 (-0.45 to -0.25)	<0.001	-0.36 (-0.46 to -0.26)	<0.001	-0.56 (-0.68 to -0.44)	<0.001
P for trend		<0.001		<0.001		<0.001

Model 1 was a crude model. Model 2 further adjusted for age, gender, race/ethnicity, educational level, and PIR based on Model 1. Model 3 further adjusted for dietary energy intake based on Model 2.

MD, mean difference; CI, confidence intervals; OBS, oxidative balance score.

Furthermore, the degree of insulin resistance was also evaluated through the HOMA-IR, with calculations available for 5,378 out of the total 11,171 individuals possessing complete data. Increased OBS was related to lower HOMA-IR (adjusted MD, -0.53; 95% CI: -0.71, -0.35). The adjusted MD for the highest OBS quartile was -8.62 (-12.64, -4.60) (P for trend < 0.001) (**Table S6**).

### 3.4 The association between oxidative balance score and all-cause mortality among metabolic syndrome patients

Survival status was recorded for 3,622 participants out of 3,621 MetS patients, with 181 dead cases and 3,440 alive cases. Due to the low prevalence of deaths among MetS patients (45 cases of coronary heart disease-related dead cases and 49 cases of cancer-related dead cases), we will not explore the correlation of OBS with cause-specific mortality. The weighting algorithm enabled the sampling sample to reflect the overall sample.

For MetS patients, a one-unit OBS increase was related to a significant decrease in the risk of all-cause mortality (adjusted hazard ratio (HR) 0.96; 95% CI; 0.92-0.9995). When the subjects in the lowest quartile of the OBS were served as a reference, the participants in the high quartiles had a decreased risk of all-cause mortality [3^rd^ quartile: 0.66 (0.18-0.82); 4^th^ quartile: 0.38 (0.18-0.82)] after full adjustment (**Table 4**). Both dietary OBS and lifestyle OBS played an important role, and they were both negatively related to the risk of all-cause mortality in subjects with MetS. The strength of the association between OBS and the risk of all-cause mortality among males with MetS might be pronounced, but the association was statistically significant for male participants solely in Model 1 and Model 2, but not in Model 3 (**Table 4**).

**Table 4:** Associations between oxidative balance score and the risk of all-cause mortality among metabolic syndrome patients.

	Model 1		Model 2		Model 3	
	HR (95%CI)	P value	HR (95%CI)	P value	HR (95%CI)	P value
OBS	0.96 (0.93 to 0.99)	0.021	0.96 (0.93 to 0.99)	0.007	0.96 (0.92 to 0.9995)	0.047
OBS category						
Q1	Reference	Reference	Reference	Reference	Reference	Reference
Q2	0.88 (0.52 to 1.52)	0.656	0.78 (0.45 to 1.36)	0.384	0.80 (0.46 to 1.39)	0.423
Q3	0.69 (0.38 to 1.24)	0.216	0.64 (0.36 to 1.16)	0.142	0.66 (0.35 to 1.26)	0.207
Q4	0.40 (0.19 to 0.87)	0.021	0.36 (0.17 to 0.76)	0.007	0.38 (0.18 to 0.82)	0.014
P for trend		0.012		0.004		0.016
Dietary OBS	0.95 (0.91 to 0.997)	0.036	0.95 (0.91 to 0.99)	0.017	0.95 (0.90 to 1.01)	0.105
Lifestyle OBS	0.97 (0.93 to 1.01)	0.148	0.96 (0.92 to 1.01)	0.083	0.96 (0.91 to 1.02)	0.153
Gender						
Male	0.95 (0.92 to 0.99)	0.011	0.96 (0.92 to 0.99)	0.023	0.96 (0.92 to 1.01)	0.136
Female	1.06 (0.92 to 1.20)	0.426	0.89 (0.78 to 1.02)	0.093	0.89 (0.78 to 1.02)	0.099

Model 1 was a crude model. Model 2 further adjusted for age, gender, race/ethnicity, educational level, and PIR based on Model 1. Model 3 further adjusted for dietary energy intake based on Model 2.

HR, hazard ratio; CI, confidence intervals; OBS, oxidative balance score.

### 3.5 Sensitivity analyses

**Figure 2:**
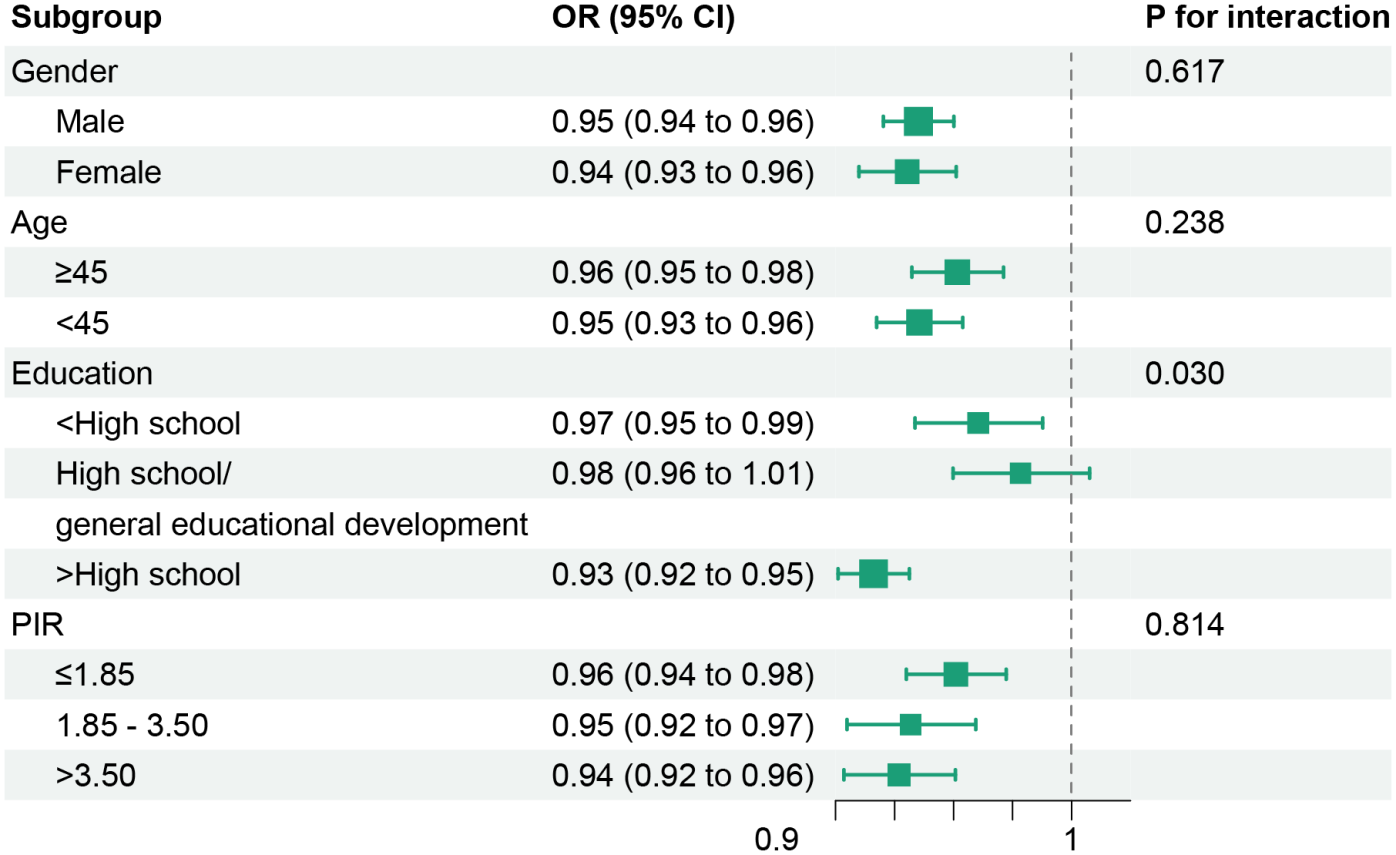
Stratified analyses of associations between oxidative balance score with metabolic syndrome risk. Models were adjusted for age, gender, race/ethnicity, educational level, PIR, and dietary energy intake (stratification factor itself was not included when stratifying). OR, odds ratio; CI, confidence intervals; PIR, poverty income ratio.

The relationships of dietary/lifestyle OBS with the susceptibility of MetS were also estimated (**Table 5**). Notably, both dietary OBS and lifestyle OBS exhibited inverse associations with the risk of MetS, alongside reductions in MetS severity score (P for interaction <0.05). Additionally, the negative correlations between OBS and the prevalence of MetS, as well as MetS severity, persisted even after conducting stratified analyses based on gender, age, education, and PIR (**Figure 2** and **Table S7**). Furthermore, the exclusion of any individual OBS component did not yield a substantial impact on the association between OBS and the risk of MetS, nor on the severity of MetS or all-cause mortality of MetS patients, as elucidated in **Table S8**.

**Table 5:** Associations between the dietary/lifestyle OBS and metabolic syndrome/metabolic syndrome severity score.

		Dietary OBS		Lifestyle OBS		
		Adjusted model	P value	Adjusted model	P value	P for interaction
MetS [OR (95%CI)]	Model 1	0.99 (0.98 to 0.9999)	0.047	0.69 (0.67 to 0.72)	<0.001	
	Model 2	0.97 (0.96 to 0.98)	<0.001	0.69 (0.66 to 0.72)	<0.001	
	Model 3	0.98 (0.97 to 0.99)	<0.001	0.69 (0.67 to 0.72)	<0.001	0.028
MetS severity score [MD (95%CI)]	Model 1	-0.01 (-0.02 to -0.01)	<0.001	-0.20 (-0.23 to -0.18)	<0.001	
	Model 2	-0.02 (-0.03 to -0.01)	<0.001	-0.20 (-0.23 to -0.18)	<0.001	
	Model 3	-0.01 (-0.02 to -0.002)	0.011	-0.20 (-0.22 to -0.18)	<0.001	0.005

Model 1 was adjusted for age, gender, race/ethnicity, educational level, and PIR. Model 2 further adjusted for dietary energy intake. Model 3 further adjusted for lifestyle OBS (or dietary OBS) based on model 2.

OR, odds ratio; MD, mean difference; CI, confidence intervals; OBS, oxidative balance score.

## 4 Discussion

In the present study, a range of models was employed to analyze data containing 11,171 participants of NHANES. We examined the relationships of OBS with the risk of MetS, MetS severity, and all-cause mortality of MetS patients. Elevated OBS was linked to a reduced risk of MetS, lower MetS severity, and diminished all-cause mortality of MetS patients. Adherence to an antioxidant diet and lifestyle would be beneficial to MetS patients. The implications of these findings underscore the potential benefits of adopting antioxidant-rich diets and lifestyles for individuals afflicted with MetS. The study underscores the significance of advocating for healthy antioxidant practices and dietary habits as integral strategies for the prevention and management of MetS.

One previous cross-sectional study explored the correlation between OBS and MetS risk in Iranian adults (27). This population-based cross-sectional study by Noruzi et al. (847 participants) exhibited no significant association between OBS and MetS (27). This OBS was derived by summing tertile scores of only thirteen pro-/antioxidant components. However, the components were different from those of our OBS. In addition to alcohol consumption, our composition includes a new selection of six components (riboflavin (38), niacin (39), vitamin B6 (40), vitamin B12 (41), magnesium (42), and copper (43)) in this study. Notably, these six components were chosen based on their relationship to both redox and MetS. Previous nhanes-based studies have revealed that increased intake of niacin and vitamin B6 were linked with reduced risk of MetS (44). Vitamin B2 deficiency consequence of insulin resistance and MetS (45). The results based on the CARDIA study indicated that both dietary consumption of vitamins B6 and B12 were negatively related to the incidence of MetS, and serum concentrations of them exhibited a similar pattern (46). In a prospective Chinese cohort study, it was shown that dietary magnesium consumption was inversely linked with MetS (47). One meta-analysis indicated an inverse correlation between dietary copper and MetS (48). Our study built on these previous findings by utilizing a different set of components to derive OBS and investigating its relationship with MetS, MetS severity, and all-cause mortality in a large NHANES-based sample.

In support of our findings, an observational study of 6,400 Koreans aged >40 years revealed a tight correlation between OBS and MetS risk (49). The study also demonstrated that subjects with the highest OBS quartile had a decreased risk for MetS than those with the lowest quartile (49). However, the OBS components included in this Korean cohort were different from ours, which included only 7 components (iron, β-carotene, vitamins C, retinol, smoking, alcohol consumption and physical activity). Moreover, we conducted a comprehensive assessment of outcomes, encompassing both the severity of MetS and all-cause mortality among MetS individuals. Furthermore, through evaluating the antioxidant status of Thai subjects with MetS, we could find that an alteration in antioxidant status was associated with MetS (50). Our results, together with these previous findings, underscore the importance of maintaining a balance between pro-oxidants and antioxidants in preventing and managing MetS patients.

Our results also exhibited that higher OBS was related to lower MetS severity. The degree of oxidative stress depends on the severity of MetS (51). Previous studies have shown that participants with higher MetS severity scores tended to have less physical activity, a pro-inflammatory dietary pattern, and lower adherence to the Mediterranean diet (52). However, a study that included 63 patients with MetS indicated no notable correlations between MetS severity and spot oxidative stress or exercise-induced oxidative stress biomarkers (53). This may be because the isolated contribution of a single component is difficult to ascertain. The imbalance between antioxidants and pro-oxidants is regarded as playing an essential role in both MetS and its constituent disorders (18).

Interestingly, for MetS participants, OBS was negatively associated with all-cause mortality, but the association was tighter in male subjects than female subjects. Previous investigations have revealed that males with MetS have a higher mortality risk than females (54, 55). Male subjects tended to have a higher prevalence of smoking, and smoking played an essential role in interpreting the gender difference in mortality (56). The correlation between MetS and unfavorable prognosis was likely influenced by a variety of controllable and uncontrollable factors (57). Our study suggests that adherence to an antioxidant diet and lifestyle might be beneficial to reducing all-cause mortality in individuals with MetS, particularly in males.

Our study had several strengths. Firstly, we employed the use of OBS, which integrated several dietary and lifestyle components into a comprehensive score, to better capture the intricate relationships among various factors associated with MetS. Moreover, a wide range of commonly used models were utilized to investigate not only the association of OBS with the risk of MetS but also the associations of OBS with MetS severity and all-cause mortality of MetS patients. Furthermore, the sensitivity analyses warranted the robustness of the associations.

Our study also had several limitations. Firstly, due to the utilization of only sixteen nutrients and four lifestyle factors being employed, our ability to acquire more accurate OBS compositions is probably limited. Considering that composite measures may provide a more precise representation of health outcomes in comparison to individual pro-oxidant/antioxidant exposure (58, 59), the OBS compositions are supposed to use a richer set of features, such as flavonoids, medication type, and additional information on diet and lifestyle. Secondly, OBS is unable to assess threshold effects and does not respond to dynamic alterations in antioxidant and pro-oxidant states. Thirdly, although our study included 11,171 participants, our analysis of mortality was limited by the relatively small number of deaths (n=181) among MetS patients. The effectiveness of OBS should be validated in larger data sets. Furthermore, the lack of biomarkers of oxidative stress in this study prevented us from verifying the effectiveness of OBS for oxidative balance assessment. Finally, in the examination concerning the relationship between OBS and MetS risk, as well as MetS severity, the outcomes attained solely indicated correlations between these factors due to the utilization of cross-sectional data.

Despite this limitation, we still found that the relationship between MetS and OBS was robust. In addition, although we were careful to perform sensitivity analyses to validate each model. This feature of NHANES’ complex sampling design enables our results to be extended to all noninstitutionalized resident adults in the US. Furthermore, it was necessary to confirm these correlations in additional retrospective and prospective populations, because we could not determine whether it was the same in other populations of different ethnicity or other cohorts.

## 5 Conclusion

This study illustrates inverse associations between OBS and the risk of MetS, as well as MetS severity and all-cause mortality of MetS patients. Higher OBS indicates greater exposure to antioxidants. This study advocates that an antioxidant diet and lifestyle contribute to the well-being of individuals with MetS. Our study underscores the pivotal role of antioxidant exposure in effectively managing MetS patients, thereby proposing a potential strategy to enhance clinical outcomes in this population. Future randomized controlled trials are necessary to firmly establish these findings and investigate the clinical effectiveness of antioxidant interventions among MetS management.

## Data availability statement

Publicly available datasets were analyzed in this study. This data can be found here: NHANES website (https://www.cdc.gov/nchs/nhanes/index.htm). The original contributions presented in the study are included in the article/**Supplementary Material**. Further inquiries can be directed to the corresponding author.

## Ethics statement

The studies involving humans were approved by National Center for Health Statistics Ethics Review Board (Protocol Number: Protocol #2005-06; Protocol #2011-17). The studies were conducted in accordance with the local legislation and institutional requirements. The participants provided their written informed consent to participate in this study. This study involved secondary data analysis of the National Health and Nutrition Examination Survey, and this study we conducted was exempt from institutional review for this reason.

## Author contributions

Conceptualities, funding acquisition, and writing-review and editing, CC and RY; methodology, software, and writing-original draft preparation, ZX; validation, data curation and writing-review and editing, XL and WC; writing-review and editing, LW. All authors contributed to the article and approved the submitted version.
