# Corrigendum: Oxidative balance score was negatively associated with the risk of metabolic syndrome, metabolic syndrome severity, and all-cause mortality of patients with metabolic syndrome

**DOI:** 10.3389/fendo.2025.1564193

**Published:** 2025-02-12

**Authors:** Zhixiao Xu, Xiong Lei, Weiwei Chu, Luoqi Weng, Chengshui Chen, Ran Ye

**Affiliations:** ^1^ Department of Ultrasonography, The Second Affiliated Hospital and Yuying Children’s Hospital of Wenzhou Medical University, Wenzhou, China; ^2^ Department of Pulmonary and Critical Care Medicine, The First Affiliated Hospital of Wenzhou Medical University, Wenzhou, China; ^3^ Department of Pulmonary and Critical Care Medicine, The Lu’an People’s Hospital of Anhui Province, The Lu’an Hospital Affiliated to Anhui Medical University, Lu’an, China; ^4^ Key Laboratory of Interventional Pulmonology of Zhejiang Province, The First Affiliated Hospital of Wenzhou Medical University, Wenzhou, China; ^5^ The Quzhou Affiliated Hospital of Wenzhou Medical University, Quzhou People’s Hospital, Quzhou, China

**Keywords:** oxidative stress, metabolic syndrome, lifestyles, diet, mortality

In the published article, there was an error in the order of the affiliations. Instead of “Zhixiao Xu^1^, Xiong Lei^1^, Weiwei Chu^2^, Luoqi Weng^1^, Chengshui Chen^1,3,4^^*^^†^ and Ran Ye^5^^*^^†^.

1 Department of Pulmonary and Critical Care Medicine, The First Affiliated Hospital of Wenzhou Medical University, Wenzhou, China.

2 Department of Pulmonary and Critical Care Medicine, The Lu’an People’s Hospital of Anhui Province, The Lu ‘an Hospital Affiliated to Anhui Medical University, Lu’an, China.

3 Key Laboratory of Interventional Pulmonology of Zhejiang Province, The First Affiliated Hospital of Wenzhou Medical University, Wenzhou, China.

4 The Quzhou Affiliated Hospital of Wenzhou Medical University, Quzhou People’s Hospital, Quzhou, China.

5 Department of Ultrasonography, The Second Affiliated Hospital and Yuying Children’s Hospital of Wenzhou Medical University, Wenzhou, China.”, it should be “Zhixiao Xu^2^, Xiong Lei^2^, Weiwei Chu^3^, Luoqi Weng^2^, Chengshui Chen^2,4,5^^*^^†^ and Ran Ye^1^^*^^†^.

1 Department of Ultrasonography, The Second Affiliated Hospital and Yuying Children’s Hospital of Wenzhou Medical University, Wenzhou, China.

2 Department of Pulmonary and Critical Care Medicine, The First Affiliated Hospital of Wenzhou Medical University, Wenzhou, China.

3 Department of Pulmonary and Critical Care Medicine, The Lu’an People’s Hospital of Anhui Province, The Lu ‘an Hospital Affiliated to Anhui Medical University, Lu’an, China.

4 Key Laboratory of Interventional Pulmonology of Zhejiang Province, The First Affiliated Hospital of Wenzhou Medical University, Wenzhou, China.

5 The Quzhou Affiliated Hospital of Wenzhou Medical University, Quzhou People’s Hospital, Quzhou, China.”

The authors apologize for this error and state that this does not change the scientific conclusions of the article in any way. The original article has been updated.

