# Supplementary material for: Oxidative balance score was negatively associated with the risk of metabolic syndrome, metabolic syndrome severity, and all-cause mortality of patients with metabolic syndrome
Source: Front Endocrinol (Lausanne). 2024 Jan 12;14:1233145. doi: 10.3389/fendo.2023.1233145 (PMC10811064; doi:10.3389/fendo.2023.1233145)
Supplement: Supplementary file 1 [file Table_1.docx]

Supplementary Material

Oxidative balance score was negatively associated with the risk of metabolic syndrome, metabolic syndrome severity, and all-cause mortality of patients with metabolic syndrome

Zhixiao Xu, Xiong Lei, Weiwei Chu, Luoqi Weng, Chengshui Chen^†*^, Ran Ye ^†*^

*** Correspondence:** †These authors have contributed equally to this work. Chengshui Chen, E-mail address: chenchengshui@wmu.edu.cn. Ran Ye, E-mail address: ranyewenz@163.com.

Table S1. Oxidative balance score (OBS) items and score assignment

| OBS components | Property | Male | | | Female | | |
| --- | --- | --- | --- | --- | --- | --- | --- |
|  |  | 0 | 1 | 2 | 0 | 1 | 2 |
| Dietary OBS components | |  |  |  |  |  |  |
| Dietary fiber (g/d) | A | <13.55 | 13.55-20.40 | ≥20.40 | <11.85 | 11.85-17.95 | ≥17.95 |
| Carotene (RE/d) | A | <595.50 | 595.50-1843.00 | ≥1843.00 | <660.50 | 660.50-2276.50 | ≥2276.50 |
| Riboflavin (mg/d) | A | <1.89 | 1.89-2.71 | ≥2.71 | <1.50 | 1.50-2.12 | ≥2.12 |
| Niacin (mg/d) | A | <25.13 | 25.13-34.51 | ≥34.51 | <17.53 | 17.53-24.29 | ≥24.29 |
| Vitamin B6 (mg/d) | A | <1.86 | 1.86-2.73 | ≥2.73 | <1.36 | 1.36-1.99 | ≥1.99 |
| Total folate (mcg/d) | A | <343.00 | 343.00-500.50 | ≥500.50 | <267.50 | 267.50-394.50 | ≥394.50 |
| Vitamin B12 (mcg/d) | A | <3.87 | 3.87-6.51 | ≥6.51 | <2.67 | 2.67-4.58 | ≥4.58 |
| Vitamin C (mg/d) | A | <37.85 | 37.085-96.35 | ≥96.35 | <38.60 | 38.60-86.15 | ≥86.15 |
| Vitamin E (ATE) (mg/d) | A | <6.58 | 6.58-10.16 | ≥10.16 | <5.74 | 5.74-8.77 | ≥8.77 |
| Calcium (mg/d) | A | <812.50 | 812.50-1230.00 | ≥1230.00 | <670.00 | 670.00-990.00 | ≥990.00 |
| Magnesium (mg/d) | A | <276.50 | 276.500-377.00 | ≥377.00 | <226.50 | 226.50-311.00 | ≥311.00 |
| Zinc (mg/d) | A | <10.40 | 10.40-14.88 | ≥14.88 | <7.59 | 7.59-10.66 | ≥10.66 |
| Copper (mg/d) | A | <1.07 | 1.07-1.51 | ≥1.51 | <0.91 | 0.91-1.27 | ≥1.27 |
| Selenium (mcg/d) | A | <109.70 | 109.70-150.55 | ≥150.55 | <77.45 | 77.45-108.35 | ≥108.35 |
| Total fat (g/d) | P | ≥105.93 | 74.87-105.93 | <74.87 | ≥79.69 | 56.24-79.69 | <56.24 |
| Iron (mg/d) | P | ≥18.21 | 12.84-18.21 | <12.84 | ≥14.04 | 9.91-14.04 | <9.91 |
| Lifestyle OBS components |  |  |  |  |  |  |  |
| Physical activity (MET-minute/week) | A | <1920 | 1920-6060 | ≥6060 | <1080 | 1080-3120 | ≥3120 |
| Acoholic drinks at past 12 months (drink/d) | P | >3 | 2-3 | ≤2 | >2 | 1-2 | ≤1 |
| Body mass index (kg/m2) | P | ≥30.05 | 25.71-30.05 | <25.71 | ≥29.90 | 24.00-29.90 | <24.00 |
| Cotinine (ng/mL) | P | ≥3.070 | 0.022-3.070 | <0.022 | ≥0.094 | 0.011-0.094 | <0.011 |

OBS: oxidative balance score; A: antioxidant; P: prooxidant; RE: retinol equivalent; ATE: alpha-tocopherol equivalent; MET: metabolic equivalent.

Table S2. Dietary and lifestyle components included in each a priori oxidative balance score related to cardiometabolic diseases

|  | Noruzi et al., 2021(1) | Cheng et al., 2023(2) | Lee et al., 2022; Son et al., 2023(3, 4) | Kong et al., 2015(5) | Wang et al., 2023(6) | Liu et al., 2023(7) | Talavera-Rodriguez et al., 2023(8) | Ilori et al., 2017(9) | Lee and Park, 2017(10) |
| --- | --- | --- | --- | --- | --- | --- | --- | --- | --- |
| **OBS components** |  |  |  |  |  |  |  |  |  |
| **Dietary OBS components** |  |  |  |  |  |  |  |  |  |
| Dietary fiber |  | √ |  |  | √ |  |  |  |  |
| Carotene | √ | √ | √ |  | √ |  | √ | √ | √ |
| Riboflavin |  | √ |  |  | √ |  |  |  |  |
| Niacin |  | √ |  |  | √ |  |  |  |  |
| Vitamin B6 |  | √ |  |  | √ |  |  |  |  |
| Total folate |  | √ |  |  | √ |  |  |  |  |
| Vitamin B12 |  | √ |  |  | √ |  |  |  |  |
| Vitamin C | √ | √ | √ | √ | √ | √ | √ | √ | √ |
| Vitamin E | √ | √ | √ |  | √ | √ | √ | √ | √ |
| Calcium |  | √ |  |  | √ |  |  |  |  |
| Magnesium |  | √ |  |  | √ |  |  |  |  |
| Zinc |  | √ | √ |  | √ |  | √ |  |  |
| Copper |  | √ | √ |  | √ |  |  |  |  |
| Selenium | √ | √ | √ | √ | √ |  | √ |  |  |
| Total fat |  | √ |  |  | √ |  |  |  |  |
| Iron | √ | √ | √ | √ | √ |  | √ | √ | √ |
| **Lifestyle OBS components** |  |  |  |  |  |  |  |  |  |
| Physical activity |  | √ | √ |  |  | √ | √ |  | √ |
| Alcohol | √ | √ | √ | √ | √ | √ | √ | √ | √ |
| Body mass index |  |  | √ |  |  | √ | √ |  |  |
| Cotinine/smoking | √ | √ | √ | √ |  | √ | √ |  | √ |
| Other OBS components | Aspirin and NSAID use; PUFA; Lutein; Lycopene | β-cryptoxanthin; Lycopene; Lutein + zeaxanthin | Saturated fatty acid; Omega-6 PUFA intake; Omega-3 PUFA intake | PUFA; Lycopene; Lutein; β-cryptoxanthin; Regular aspirin use; Regular NSAID use |  | Aspirin and NSAID use; Serum ferritin; β-cryptoxanthin; zeaxanthin; Plasma ω-3/ω-6 fatty acid | Polyphenols, Total antioxidant capacity | PUFA; Lycopene; Lutein; Cryptoxanthin; Statins | Retinol |

PUFA, Polyunsaturated fatty acid; NSAID, non-steroidal anti-inflammatory drug; OBS: oxidative balance score

Table S3. The National Cholesterol Education Program Adult Treatment Panel III (NCEP ATP III) definition for metabolic syndrome

| Risk factors | Cut-off points | And/or drug treatment |
| --- | --- | --- |
| Abdominal obesity (waist circumference) | ≥ 102 cm in men  ≥ 88 cm in women | - |
| Hypertriglyceridemia | serum triglyceride ≥150 mg/dL (1.7 mmol/L) | drug treatment for elevated triglycerides |
| Low high-density cholesterols (HDL) | serum high density lipoprotein cholesterol (HDL-C) < 40 mg/dL in men  serum HDL-C < 50 mg/dL in women | drug treatment for low HDL-C, fibrates, and/or nicotinic acid |
| Hypertension | systolic blood pressure (SBP) ≥ 130 mmHg or diastolic blood pressure (DBP) ≥ 85 mmHg | receiving anti-hypertensive treatment, or diagnosed with hypertension by a doctor |
| Hyperglycemia | Fasting glucose ≥100 mg/dL | receiving anti-hyperglycemic treatments, or taking insulin, or being diagnosed with diabetes |

Table S4. Distribution for oxidative balance score

|  | Oxidative Balance Score | | | |
| --- | --- | --- | --- | --- |
|  | Mean ±SD | P value | Median [IQR] | P value |
| Age (year) |  | <0.001 |  | <0.001 |
| <45 | 19.64±7.12 |  | 20 [14, 25] |  |
| ≥45 | 20.53±6.94 |  | 21 [15, 26] |  |
| Gender |  | 0.02 |  | 0.034 |
| Male | 20.19±7.00 |  | 20 [15, 26] |  |
| Female | 19.83±7.13 |  | 20 [14, 26] |  |
| Race/ethnicity |  | <0.001 |  | <0.001 |
| Mexican American | 19.63 ±6.85 |  | 20 [14, 25] |  |
| Other Hispanic | 19.81±7.16 |  | 20 [14, 25] |  |
| Non-Hispanic White | 20.44±7.04 |  | 21 [15, 26] |  |
| Non-Hispanic Black | 17.12±6.91 |  | 16 [11, 22] |  |
| Other Race - Including Multi-Racial | 20.21±6.72 |  | 20 [15, 25] |  |
| Educational level |  | <0.001 |  | <0.001 |
| <High school | 17.56±6.81 |  | 17 [12, 23] |  |
| High school/general educational development | 18.20±6.94 |  | 18 [12, 24] |  |
| >High school | 20.97±6.93 |  | 21 [16, 26] |  |
| Metabolic syndrome |  | <0.001 |  | <0.001 |
| No | 20.33±7.15 |  | 21 [15, 26] |  |
| Yes | 19.32±6.81 |  | 19 [14, 25] |  |
| Abdominal obesity |  | <0.001 |  | <0.001 |
| No | 21.05±7.01 |  | 22 [16, 27] |  |
| Yes | 18.99±6.96 |  | 19 [13, 24] |  |
| Hypertriglyceridemia |  | 0.024 |  | 0.022 |
| No | 20.17±7.15 |  | 21 [15, 26] |  |
| Yes | 19.77±6.90 |  | 20 [14, 25] |  |
| Low high-density cholesterols |  | <0.001 |  | <0.001 |
| No | 20.40±7.09 |  | 21 [15, 26] |  |
| Yes | 19.24±6.92 |  | 19 [14, 25] |  |
| Hypertension |  | 0.004 |  | 0.004 |
| No | 20.22±7.16 |  | 21 [15, 26] |  |
| Yes | 19.57±6.81 |  | 20 [14, 25] |  |
| Hyperglycemia |  | 0.125 |  | 0.122 |
| No | 20.10±7.13 |  | 20 [15, 26] |  |
| Yes | 19.75±6.80 |  | 20 [14, 25] |  |
| Final mortality status |  | 0.024 |  | 0.021 |
| Alive | 19.38±6.80 |  | 20 [14, 25] |  |
| Deceased | 17.68±6.75 |  | 17 [12, 22.57] |  |

Table S5. Association of oxidative balance score with metabolic syndrome components

|  | Continue | Q1 | Q2 | Q3 | Q4 | P for trend |
| --- | --- | --- | --- | --- | --- | --- |
| Abdominal obesity | |  |  |  |  |  |
| Model 1 | 0.95 (0.94 to 0.96) | Reference | 0.70 (0.59 to 0.83) | 0.62 (0.53 to 0.73) | 0.40 (0.34 to 0.47) | <0.001 |
| Model 2 | 0.93 (0.92 to 0.93) | Reference | 0.60 (0.50 to 0.72) | 0.47 (0.39 to 0.55) | 0.26 (0.22 to 0.31) | <0.001 |
| Hypertriglyceridemia | |  |  |  |  |  |
| Model 1 | 0.98 (0.97 to 0.99) | Reference | 0.999 (0.85 to 1.17) | 0.84 (0.73 to 0.98) | 0.75 (0.63 to 0.89) | <0.001 |
| Model 2 | 0.96 (0.95 to 0.97) | Reference | 0.89 (0.76 to 1.04) | 0.68 (0.58 to 0.79) | 0.54 (0.44 to 0.67) | <0.001 |
| Low high-density cholesterols | |  |  |  |  |  |
| Model 1 | 0.98 (0.97 to 0.98) | Reference | 0.95 (0.82 to 1.10) | 0.81 (0.68 to 0.98) | 0.68 (0.57 to 0.80) | <0.001 |
| Model 2 | 0.96 (0.95 to 0.97) | Reference | 0.88 (0.76 to 1.02) | 0.70 (0.57 to 0.86) | 0.54 (0.44 to 0.67) | <0.001 |
| Hypertension | |  |  |  |  |  |
| Model 1 | 0.98 (0.97 to 0.99) | Reference | 1.01 (0.82 to 1.24) | 0.85 (0.69 to 1.04) | 0.70 (0.58 to 0.86) | <0.001 |
| Model 2 | 0.96 (0.95 to 0.97) | Reference | 0.92 (0.74 to 1.14) | 0.71 (0.56 to 0.91) | 0.55 (0.42 to 0.71) | <0.001 |
| Hyperglycemia | |  |  |  |  |  |
| Model 1 | 0.99 (0.98 to 0.998) | Reference | 1.12 (0.95 to 1.32) | 1.01 (0.84 to 1.20) | 0.87 (0.71 to 1.06) | 0.073 |
| Model 2 | 0.98 (0.97 to 0.99) | Reference | 1.06 (0.89 to 1.26) | 0.91 (0.74 to 1.12) | 0.75 (0.59 to 0.96) | 0.008 |

Model 1 adjusted for age, gender, race/ethnicity, educational level, and PIR. Model 2 further adjusted for dietary energy intake based on Model 1.

OR, odds ratio; CI, confidence intervals; OBS, oxidative balance score.

Table S6. Influence of oxidative balance score on Homeostatic Model Assessment for insulin resistance

|  | Model 1 | |  | Model 2 | |  | Model 3 | |
| --- | --- | --- | --- | --- | --- | --- | --- | --- |
|  | MD (95%CI) | P value |  | MD (95%CI) | P value |  | MD (95%CI) | P value |
| OBS | -0.37 (-0.52 to -0.22) | <0.001 |  | -0.36 (-0.53 to -0.18) | <0.001 |  | -0.53 (-0.71 to -0.35) | <0.001 |
| OBS per SD | -2.61 (-3.68 to -1.54) | <0.001 |  | -2.53 (-3.75 to -1.31) | <0.001 |  | -3.78 (-5.05 to -2.50) | <0.001 |
| OBS category | |  |  |  |  |  |  |  |
| Q1 | Reference | Reference |  | Reference | Reference |  | Reference | Reference |
| Q2 | -1.90 (-5.16 to 1.37) | 0.251 |  | -1.85 (-5.25 to 1.56) | 0.284 |  | -2.82 (-6.46 to 0.81) | 0.126 |
| Q3 | -4.54 (-7.40 to -1.68) | 0.002 |  | -4.45 (-7.53 to -1.37) | 0.005 |  | -6.28 (-9.59 to -2.97) | <0.001 |
| Q4 | -6.23 (-9.43 to -3.03) | <0.001 |  | -5.86 (-9.53 to -2.20) | 0.002 |  | -8.62 (-12.64 to -4.60) | <0.001 |
| P for trend | | <0.001 |  |  | <0.001 |  |  | <0.001 |

Model 1 was a crude model. Model 2 further adjusted for age, gender, race/ethnicity, educational level, and PIR based on Model 1. Model 3 further adjusted for dietary energy intake based on Model 2.

MD, mean difference; CI, confidence intervals; OBS, oxidative balance score.

Table S7. Stratified analyses of associations between oxidative balance score with metabolic syndrome severity

| Subgroup | MD (95%CI) | P value | P for interaction |
| --- | --- | --- | --- |
| Gender |  |  | <0.001 |
| Male | -0.03 (-0.04 to -0.02) | <0.001 |  |
| Female | -0.03 (-0.04 to -0.03) | <0.001 |  |
| Age |  |  | 0.722 |
| ≥45 | -0.03 (-0.04 to -0.02) | <0.001 |  |
| <45 | -0.03 (-0.04 to -0.02) | <0.001 |  |
| Education |  |  | 0.841 |
| <High school | -0.02 (-0.04 to -0.004) | 0.019 |  |
| High school/general educational development | -0.03 (-0.04 to -0.03) | <0.001 |  |
| >High school | -0.03 (-0.05 to -0.02) | <0.001 |  |
| PIR |  |  | <0.001 |
| ≤1.85 | -0.02 (-0.03 to -0.02) | <0.001 |  |
| 1.85 - 3.50 | -0.04 (-0.06 to -0.02) | <0.001 |  |
| >3.50 | -0.03 (-0.04 to -0.02) | <0.001 |  |

Model 1 adjusted for age, gender, race/ethnicity, educational level, and PIR. Model 2 further adjusted for dietary energy intake based on Model 1.

MD, mean difference; CI, confidence intervals; OBS, oxidative balance score.

Table S8. Sensitivity analyses to evaluate the impact of individual oxidative balance score components on the oxidative balance score

|  | MetS | |  | MetS severity score | |  | Risk of all-cause mortality | |
| --- | --- | --- | --- | --- | --- | --- | --- | --- |
|  | OR (95%CI) | P value |  | MD (95%CI) | P value |  | HR (95%CI) | P value |
| OBS excluding dietary fiber | 0.94 (0.93 to 0.95) | <0.001 |  | -0.04 (-0.04 to -0.03) | <0.001 |  | 0.96 (0.92 to 0.9996) | 0.048 |
| OBS excluding carotene | 0.94 (0.93 to 0.96) | <0.001 |  | -0.03 (-0.04 to -0.03) | <0.001 |  | 0.96 (0.92 to 1.006) | 0.089 |
| OBS excluding riboflavin | 0.94 (0.93 to 0.95) | <0.001 |  | -0.04 (-0.04 to -0.03) | <0.001 |  | 0.96 (0.92 to 0.998) | 0.039 |
| OBS excluding niacin | 0.94 (0.93 to 0.95) | <0.001 |  | -0.04 (-0.04 to -0.03) | <0.001 |  | 0.95 (0.92 to 0.995) | 0.027 |
| OBS excluding vitamin B6 | 0.94 (0.93 to 0.95) | <0.001 |  | -0.04 (-0.04 to -0.03) | <0.001 |  | 0.96 (0.92 to 1.00) | 0.052 |
| OBS excluding total folate | 0.94 (0.93 to 0.95) | <0.001 |  | -0.04 (-0.04 to -0.03) | <0.001 |  | 0.96 (0.92 to 1.005) | 0.086 |
| OBS excluding vitamin B12 | 0.94 (0.93 to 0.95) | <0.001 |  | -0.04 (-0.04 to -0.03) | <0.001 |  | 0.95 (0.91 to 0.997) | 0.038 |
| OBS excluding vitamin C | 0.94 (0.93 to 0.96) | <0.001 |  | -0.04 (-0.04 to -0.03) | <0.001 |  | 0.96 (0.92 to 1.006) | 0.089 |
| OBS excluding vitamin E | 0.94 (0.93 to 0.95) | <0.001 |  | -0.03 (-0.04 to -0.03) | <0.001 |  | 0.96 (0.92 to 1.003) | 0.067 |
| OBS excluding calcium | 0.94 (0.93 to 0.95) | <0.001 |  | -0.04 (-0.04 to -0.03) | <0.001 |  | 0.95 (0.91 to 0.992) | 0.021 |
| OBS excluding magnesium | 0.94 (0.93 to 0.95) | <0.001 |  | -0.04 (-0.04 to -0.03) | <0.001 |  | 0.96 (0.92 to 0.997) | 0.037 |
| OBS excluding zinc | 0.94 (0.93 to 0.95) | <0.001 |  | -0.04 (-0.04 to -0.03) | <0.001 |  | 0.96 (0.92 to 0.997) | 0.036 |
| OBS excluding copper | 0.94 (0.93 to 0.95) | <0.001 |  | -0.04 (-0.04 to -0.03) | <0.001 |  | 0.95 (0.91 to 0.995) | 0.028 |
| OBS excluding selenium | 0.94 (0.93 to 0.95) | <0.001 |  | -0.04 (-0.04 to -0.03) | <0.001 |  | 0.96 (0.92 to 0.995) | 0.028 |
| OBS excluding total fat | 0.95 (0.94 to 0.96) | <0.001 |  | -0.03 (-0.04 to -0.03) | <0.001 |  | 0.96 (0.92 to 0.9996) | 0.048 |
| OBS excluding iron | 0.95 (0.94 to 0.96) | <0.001 |  | -0.03 (-0.04 to -0.02) | <0.001 |  | 0.96 (0.93 to 1.001) | 0.056 |
| OBS excluding physical activity | 0.95 (0.94 to 0.96) | <0.001 |  | -0.03 (-0.04 to -0.03) | <0.001 |  | 0.96 (0.92 to 1.003) | 0.067 |
| OBS excluding alcohol | 0.94 (0.93 to 0.96) | <0.001 |  | -0.03 (-0.04 to -0.03) | <0.001 |  | 0.96 (0.92 to 1.003) | 0.069 |
| OBS excluding body mass index | 0.97 (0.96 to 0.98) | <0.001 |  | -0.02 (-0.03 to -0.01) | <0.001 |  | 0.96 (0.92 to 1.002) | 0.062 |
| OBS excluding cotinine | 0.94 (0.93 to 0.96) | <0.001 |  | -0.03 (-0.04 to -0.03) | <0.001 |  | 0.96 (0.92 to 1.001) | 0.057 |

Models adjusted for age, gender, race/ethnicity, educational level, PIR, and dietary energy intake.

OR, odds ratio; MD, mean difference; HR, hazard ratio; CI, confidence intervals; OBS, oxidative balance score.

**Reference:**

1. Noruzi Z, Jayedi A, Farazi M, Asgari E, Dehghani Firouzabadi F, Akbarzadeh Z, et al. Association of Oxidative Balance Score with the Metabolic Syndrome in a Sample of Iranian Adults. *Oxid Med Cell Longev* (2021) 2021:5593919. Epub 2021/07/01. doi: 10.1155/2021/5593919.

2. Cheng S, Han Y, Jiang L, Lan Z, Liao H, Guo J. Associations of Oxidative Balance Score and Visceral Adiposity Index with Risk of Ischaemic Heart Disease: A Cross-Sectional Study of Nhanes, 2005-2018. *BMJ Open* (2023) 13(7):e072334. Epub 2023/07/15. doi: 10.1136/bmjopen-2023-072334.

3. Lee JH, Son DH, Kwon YJ. Association between Oxidative Balance Score and New-Onset Hypertension in Adults: A Community-Based Prospective Cohort Study. *Front Nutr* (2022) 9:1066159. Epub 2023/01/03. doi: 10.3389/fnut.2022.1066159.

4. Son DH, Lee HS, Seol SY, Lee YJ, Lee JH. Association between the Oxidative Balance Score and Incident Chronic Kidney Disease in Adults. *Antioxidants (Basel)* (2023) 12(2). Epub 2023/02/26. doi: 10.3390/antiox12020335.

5. Kong SY, Goodman M, Judd S, Bostick RM, Flanders WD, McClellan W. Oxidative Balance Score as Predictor of All-Cause, Cancer, and Noncancer Mortality in a Biracial Us Cohort. *Ann Epidemiol* (2015) 25(4):256-62.e1. Epub 2015/02/17. doi: 10.1016/j.annepidem.2015.01.004.

6. Wang X, Hu J, Liu L, Zhang Y, Dang K, Cheng L, et al. Association of Dietary Inflammatory Index and Dietary Oxidative Balance Score with All-Cause and Disease-Specific Mortality: Findings of 2003-2014 National Health and Nutrition Examination Survey. *Nutrients* (2023) 15(14). Epub 2023/07/29. doi: 10.3390/nu15143148.

7. Liu J, He L, Wang A, Lv Y, He H, Wang C, et al. Oxidative Balance Score Reflects Vascular Endothelial Function of Chinese Community Dwellers. *Front Physiol* (2023) 14:1076327. Epub 2023/05/04. doi: 10.3389/fphys.2023.1076327.

8. Talavera-Rodriguez I, Fernandez-Lazaro CI, Hernández-Ruiz Á, Hershey MS, Galarregui C, Sotos-Prieto M, et al. Association between an Oxidative Balance Score and Mortality: A Prospective Analysis in the Sun Cohort. *Eur J Nutr* (2023) 62(4):1667-80. Epub 2023/02/14. doi: 10.1007/s00394-023-03099-8.

9. Ilori TO, Wang X, Huang M, Gutierrez OM, Narayan KM, Goodman M, et al. Oxidative Balance Score and the Risk of End-Stage Renal Disease and Cardiovascular Disease. *Am J Nephrol* (2017) 45(4):338-45. Epub 2017/03/13. doi: 10.1159/000464257.

10. Lee HS, Park T. Pathway-Driven Approaches of Interaction between Oxidative Balance and Genetic Polymorphism on Metabolic Syndrome. *Oxid Med Cell Longev* (2017) 2017:6873197. Epub 2017/02/14. doi: 10.1155/2017/6873197.
